# Supplementary material for: Salivary proteome of a Neotropical primate: potential roles in host defense and oral food perception
Source: PeerJ. 2020 Jul 22;8:e9489. doi: 10.7717/peerj.9489 (PMC7382365; doi:10.7717/peerj.9489)
Supplement: Supplemental Information 1 [file peerj-08-9489-s001.docx]

**SUPPLEMENTARY TABLE 1:**

**Salivary proteins identified in the whole saliva of the Neotropical black howler monkey *Alouatta pigr*a (Nano LC-MS/MS).**

| Protein name | UniProtKB Accession number | Matching species | Molecular mass (kDa) | Sequence coverage (%) | Mascot score | Matched peptides |
| --- | --- | --- | --- | --- | --- | --- |
| 78 kDa glucose-regulated protein | P11021 | *Homo sapiens* | 72.288 | 51 | 587 | 29 |
| Actin gamma 1 | F6TII3 | *Macaca mulatta* | 42.276 | 6 | 32 | 1 |
| Alcohol dehydrogenase class 4 mu/sigma chain | P40394 | *Homo sapiens* | 41.454 | 11 | 55 | 1 |
| Aldehyde dehydrogenase | F7C1B5 | *Macaca mulatta* | 57.546 | 2 | 44 | 1 |
| Aldehyde dehydrogenase, dimeric NADP-preferring | P30838 | *Homo sapiens* | 50.363 | 18 | 118 | 5 |
| Aldo-keto reductase family 1 member B10 | O60218 | *Homo sapiens* | 35.997 | 3 | 55 | 1 |
| Alpha-enolase | P06733 | *Homo sapiens* | 47.139 | 43 | 350 | 12 |
| Aminopeptidase | P15144 | *Homo sapiens* | 109.471 | 5 | 105 | 4 |
| Antithrombin-III | P01008 | *Homo sapiens* | 52.569 | 7 | 42 | 2 |
| Beta-2-microglobulin | O77523 | *Alouatta seniculus* | 13.687 | 8 | 39 | 1 |
| Beta-defensin 1 | Q95M66 | *Saguinus oedipus* | 7.417 | 1 | 51 | 1 |
| Bifunctional purine biosynthesis protein PURH | P31939 | *Homo sapiens* | 64.575 | 9 | 55 | 3 |
| Biotinidase | P43251 | *Homo sapiens* | 61.093 | 7 | 73 | 2 |
| BPI fold-containing family B member 1 | Q8TDL5 | *Homo sapiens* | 52.408 | 7 | 245 | 3 |
| Calmodulin-like protein 3 | P27482 | *Homo sapiens* | 16.88 | 11 | 47 | 1 |
| Carbonic anhydrase 6 | P23280 | *Homo sapiens* | 35.345 | 13 | 332 | 3 |
| Catalase | Q2I6W4 | *Callithrix jacchus* | 59.763 | 35 | 285 | 14 |
| Cathelicidin antimicrobial peptide | Q1KLX0 | *Saguinus oedipus* | 18.775 | 7 | 47 | 1 |
| Cathepsin B | P07858 | *Homo sapiens* | 37.797 | 5 | 43 | 1 |
| Cellular retinoic acid-binding protein 2 | P29373 | *Homo sapiens* | 15.683 | 28 | 126 | 3 |
| Clusterin | P10909 | *Homo sapiens* | 52.461 | 11 | 72 | 2 |
| Collectin subfamily member 11 | F7H2F8 | *Macaca mulatta* | 28.564 | 4 | 41 | 1 |
| Complement C3 | P01024 | *Homo sapiens* | 187.03 | 5 | 151 | 4 |
| Complement C4-A | P0C0L4 | *Homo sapiens* | 192.664 | 2 | 117 | 2 |
| Cornulin | Q9UBG3 | *Homo sapiens* | 53.502 | 5 | 64 | 1 |
| CRP55 | F7FMQ2 | *Macaca mulatta* | 48.084 | 17 | 72 | 3 |
| Cystatin-A | P01040 | *Homo sapiens* | 11 | 12 | 61 | 1 |
| Cystatin-B | Q8I030 | *Pan troglodytes* | 11.147 | 24 | 196 | 2 |
| Cystatin-C | O19093 | *Saimiri sciureus* | 15.936 | 29 | 96 | 2 |
| Cystatin-D | P28325 | *Homo sapiens* | 16.07 | 14 | 115 | 2 |
| Cytokeratin-1 | F7B786 | *Macaca mulatta* | 65.17 | 30 | 413 | 16 |
| Cytokeratin-10 (Fragment) | G7PUP9 | *Macaca fascicularis* | 57.824 | 13 | 299 | 7 |
| Cytokeratin-1B | G7PID4 | *Macaca fascicularis* | 62.173 | 4 | 108 | 2 |
| Cytokeratin-2e | G7PID3 | *Macaca fascicularis* | 65.462 | 11 | 213 | 7 |
| Cytokeratin-6C | G7PIC8 | *Macaca fascicularis* | 59.85 | 6 | 110 | 3 |
| Deoxyribonuclease-1 | P24855 | *Homo sapiens* | 31.414 | 4 | 70 | 1 |
| Dermcidin | P81605 | *Homo sapiens* | 11.277 | 22 | 52 | 1 |
| Desmoplakin-3 | F7H7V0 | *Macaca mulatta* | 81.723 | 2 | 43 | 1 |
| DnaJ homolog subfamily C member 3 | F6VD19 | *Macaca mulatta* | 57.601 | 9 | 86 | 5 |
| Elongation factor 1-alpha | G7NZC8 | *Macaca fascicularis* | 50.155 | 2 | 33 | 1 |
| Elongation factor 2 | A0SXL6 | *Callithrix jacchus* | 95.25 | 13 | 46 | 1 |
| Endoplasmic reticulum lectin 1 isoform 1 | F7BCV3 | *Macaca mulatta* | 54.886 | 5 | 28 | 1 |
| Endoplasmin | F7EZT6 | *Macaca mulatta* | 92.555 | 3 | 31 | 2 |
| ER-Golgi intermediate compartment 53 kDa protein | F6SS58 | *Macaca mulatta* | 57.322 | 13 | 66 | 3 |
| ERO1-like protein alpha | Q96HE7 | *Homo sapiens* | 54.358 | 21 | 125 | 7 |
| Ezrin | P15311 | *Homo sapiens* | 69.37 | 10 | 70 | 3 |
| Fatty acid-binding protein, epidermal | Q01469 | *Homo sapiens* | 15.155 | 28 | 92 | 3 |
| Fructose-bisphosphate aldolase A | A5A6I5 | *Pan troglodytes* | 39.409 | 4 | 61 | 1 |
| Furin | G7MVY8 | *Macaca mulatta* | 86.652 | 34 | 925 | 20 |
| Galectin-7 | P47929 | *Homo sapiens* | 15.066 | 12 | 46 | 1 |
| Gamma-enolase | G7PJP0 | *Macaca fascicularis* | 51.035 | 4 | 99 | 1 |
| Gamma-glutamyl hydrolase | Q92820 | *Homo sapiens* | 35.941 | 4 | 63 | 1 |
| Gelsolin | P06396 | *Homo sapiens* | 85.644 | 14 | 113 | 4 |
| Glucose-6-phosphate isomerase | P06744 | *Homo sapiens* | 63.107 | 22 | 266 | 6 |
| Glucosidase 2 subunit beta | P14314 | *Homo sapiens* | 59.388 | 18 | 76 | 6 |
| Glutaminyl-peptide cyclotransferase | F7GTQ0 | *Macaca mulatta* | 40.908 | 4 | 61 | 1 |
| Glutaredoxin-1 | P35754 | *Homo sapiens* | 11.768 | 13 | 299 | 1 |
| Glyceraldehyde-3-phosphate dehydrogenase | P04406 | *Homo sapiens* | 36.03 | 4 | 42 | 1 |
| Guanylate-binding protein 6 | Q6ZN66 | *Homo sapiens* | 72.381 | 14 | 91 | 5 |
| Haptoglobin | P50417 | *Ateles geoffroyi* | 38.451 | 3 | 50 | 1 |
| Heat shock 70 kDa protein 1 | Q5R7D3 | *Pongo abelii* | 70.009 | 28 | 259 | 10 |
| Heat shock cognate 71 kDa protein isoform 1 | F6RLH5 | *Macaca mulatta* | 70.854 | 24 | 194 | 10 |
| Heat shock-related 70 kDa protein 2 | P54652 | *Homo sapiens* | 69.978 | 9 | 195 | 5 |
| Hemoglobin beta chain | G7PQY3 | *Macaca fascicularis* | 16.104 | 16 | 137 | 2 |
| Histidine-rich glycoprotein | P04196 | *Homo sapiens* | 59.541 | 7 | 149 | 3 |
| Histone H1c | G7P2N4 | *Macaca fascicularis* | 22.314 | 5 | 40 | 1 |
| Histone H2A (Fragment) | F6TDW8 | *Macaca mulatta* | 15.578 | 13 | 78 | 2 |
| Histone H4 | G7P2N0 | *Macaca fascicularis* | 11.36 | 10 | 74 | 1 |
| IgG mu chain C region | P01871 | *Homo sapiens* | 49.276 | 6 | 46 | 2 |
| Junction plakoglobin | P14923 | *Homo sapiens* | 81.693 | 2 | 43 | 1 |
| Keratin 14 | F7H312 | *Macaca mulatta* | 51.851 | 14 | 118 | 4 |
| Keratin, type I cytoskeletal | P02533 | *Homo sapiens* | 51.529 | 24 | 62 | 6 |
| Keratin, type I cytoskeletal 10 | P13645 | *Homo sapiens* | 58.792 | 42 | 518 | 15 |
| Keratin, type I cytoskeletal 13 | P13646 | *Homo sapiens* | 49.557 | 24 | 121 | 7 |
| Keratin, type I cytoskeletal 14 | P02533 | *Homo sapiens* | 51.529 | 12 | 172 | 5 |
| Keratin, type I cytoskeletal 9 | P35527 | *Homo sapiens* | 62.027 | 36 | 553 | 17 |
| Keratin, type II cytoskeletal 1 | P04264 | *Homo sapiens* | 65.999 | 36 | 839 | 17 |
| Keratin, type II cytoskeletal 2 | P35908 | *Homo sapiens* | 65.393 | 22 | 304 | 8 |
| Keratin, type II cytoskeletal 2 epidermal | P35908 | *Homo sapiens* | 65.393 | 11 | 173 | 5 |
| Keratin, type II cytoskeletal 4 | P19013 | *Homo sapiens* | 57.25 | 17 | 103 | 4 |
| Keratin, type II cytoskeletal 5 | P13647 | *Homo sapiens* | 62.34 | 4 | 66 | 2 |
| Keratin, type II cytoskeletal 6A | P02538 | *Homo sapiens* | 60.008 | 7 | 146 | 4 |
| Keratin, type II cytoskeletal 6B | P04259 | *Homo sapiens* | 60.03 | 10 | 58 | 2 |
| Keratin, type II cytoskeletal 74 | Q7RTS7 | *Homo sapiens* | 57.83 | 2 | 49 | 1 |
| Keratin, type II cytoskeletal 78 | Q8N1N4 | *Homo sapiens* | 56.83 | 7 | 64 | 3 |
| L-lactate dehydrogenase B chain | A5A6N7 | *Homo sapiens* | 36.629 | 7 | 44 | 1 |
| Lactoperoxidase | P22079 | *Homo sapiens* | 80.237 | 30 | 478 | 15 |
| Lactotransferrin | P02788 | *Homo sapiens* | 78.132 | 11 | 335 | 8 |
| LanC-like protein 1 | O43813 | *Homo sapiens* | 45.254 | 3 | 65 | 1 |
| Legumain | Q4R4T8 | *Macaca fascicularis* | 49.406 | 4 | 28 | 1 |
| Leukocyte elastase inhibitor | P30740 | *Homo sapiens* | 42.715 | 6 | 58 | 2 |
| Leukotriene A(4) hydrolase | F7H193 | *Macaca mulatta* | 69.302 | 6 | 85 | 2 |
| Malate dehydrogenase, mitochondrial | P40926 | *Homo sapiens* | 35.481 | 26 | 93 | 2 |
| Matrix metalloproteinase-9 | F6W5A7 | *Macaca mulatta* | 78.431 | 2 | 35 | 1 |
| Neural proliferation differentiation and control protein 1 | Q9NQX5 | *Homo sapiens* | 34.494 | 4 | 58 | 1 |
| Neural proliferation, differentiation and control 1 | F7DPX1 | *Macaca mulatta* | 34.566 | 4 | 58 | 1 |
| Neutral alpha-glucosidase AB | Q14697 | *Homo sapiens* | 106.807 | 2 | 63 | 1 |
| Nucleobindin-1 | Q02818 | *Homo sapiens* | 53.846 | 23 | 67 | 8 |
| Nucleobindin-2 | P80303 | *Homo sapiens* | 50.164 | 40 | 462 | 16 |
| Nucleoside diphosphate kinase | G7PUB9 | *Macaca fascicularis* | 19.389 | 5 | 56 | 1 |
| Nucleotide exchange factor SIL1 | Q9H173 | *Homo sapiens* | 52.052 | 6 | 65 | 2 |
| Ovostatin homolog 2 | Q6IE36 | *Homo sapiens* | 161.149 | 4 | 32 | 2 |
| Peptidyl-prolyl cis-trans isomerase A | Q6DTV9 | *Aotus trivirgatus* | 17.886 | 55 | 148 | 4 |
| Peptidyl-prolyl cis-trans isomerase B | P23284 | *Homo sapiens* | 23.728 | 12 | 125 | 2 |
| Plastin-2 | P13796 | *Homo sapiens* | 70.244 | 22 | 117 | 8 |
| Plastin-3 isoform 1 | F7GYM9 | *Macaca mulatta* | 70.766 | 8 | 126 | 3 |
| Polyubiquitin-C | G7PWZ9 | *Macaca fascicularis* | 13.068 | 13 | 33 | 1 |
| POTE ankyrin domain family member E | Q6S8J3 | *Homo sapiens* | 121.286 | 2 | 24 | 1 |
| Profilin-1 | P07737 | *Homo sapiens* | 15.045 | 51 | 178 | 5 |
| Prosaposin | P07602 | *Homo sapiens* | 58.074 | 13 | 135 | 5 |
| Protein disulfide-isomerase | P07237 | *Homo sapiens* | 57.081 | 18 | 54 | 5 |
| Protein ERGIC-53 | P49257 | *Homo sapiens* | 57.513 | 13 | 60 | 3 |
| Protein S100 | G7NUG4 | *Macaca fascicularis* | 10.173 | 13 | 46 | 1 |
| Protein S100-A6 | P06703 | *Homo sapiens* | 10.173 | 10 | 45 | 1 |
| Protein S100-A8 | P05109 | *Homo sapiens* | 10.828 | 24 | 97 | 2 |
| Putative aldo-keto reductase family 1 member C8 | Q5T2L2 | *Homo sapiens* | 14.578 | 6 | 55 | 1 |
| Putative ubiquitin-conjugating enzyme E2 N-like | Q5JXB2 | *Homo sapiens* | 17.366 | 7 | 55 | 1 |
| Putative uncharacterized protein | G7PRG1 | *Macaca fascicularis* | 22.62 | 7 | 37 | 1 |
| Putative uncharacterized protein | G7PQY2 | *Macaca fascicularis* | 16.117 | 7 | 38 | 1 |
| Putative uncharacterized protein | G7PUS5 | *Macaca fascicularis* | 51.688 | 9 | 166 | 4 |
| Putative uncharacterized protein | G7PHX5 | *Macaca fascicularis* | 11.259 | 10 | 58 | 1 |
| Putative uncharacterized protein | G8F2G9 | *Macaca fascicularis* | 27.784 | 7 | 53 | 2 |
| Putative uncharacterized protein | G7P5L7 | *Macaca fascicularis* | 68.828 | 2 | 52 | 1 |
| Putative uncharacterized protein | G7NY31 | *Macaca fascicularis* | 77.774 | 1 | 52 | 1 |
| Putative uncharacterized protein | G7PT41 | *Macaca fascicularis* | 42.173 | 3 | 44 | 1 |
| Putative uncharacterized protein | G7PGL4 | *Macaca fascicularis* | 52.596 | 2 | 38 | 1 |
| Putative uncharacterized protein 1 | G7PUF6 | *Macaca fascicularis* | 80.694 | 1 | 38 | 1 |
| Pyruvate kinase | F7F0E8 | *Macaca mulatta* | 58.044 | 17 | 89 | 5 |
| Retinol-binding protein 2 | P06768 | *Rattus norvegicus* | 15.575 | 7 | 44 | 1 |
| Ribonuclease T2 | O00584 | *Homo sapiens* | 29.462 | 6 | 47 | 1 |
| Serine/threonine-protein phosphatase 2A 65 kDa regulatory subunit A alpha isoform | F6RRV0 | *Macaca mulatta* | 65.267 | 12 | 66 | 4 |
| Serotransferrin | A5A6I6 | *Pan troglodytes* | 77.014 | 19 | 299 | 8 |
| Serpin B5 | B1MTB7 | *Callicebus moloch* | 41.981 | 3 | 58 | 1 |
| Serum albumin | F7HCH2 | *Macaca mulatta* | 68.829 | 20 | 570 | 14 |
| Sulfhydryl oxidase 1 | H2N4I1 | *Pongo abelii* | 82.752 | 6 | 112 | 3 |
| Syncytin-1 | P61561 | *Gorilla gorilla gorilla* | 59.9 | 2 | 45 | 1 |
| Thioltransferase-1 | G7P7Z2 | *Macaca fascicularis* | 11.839 | 10 | 45 | 1 |
| Thioredoxin | Q5R9M3 | *Pongo abelii* | 11.877 | 11 | 46 | 1 |
| Thrombospondin-1 | P07996 | *Homo sapiens* | 129.3 | 1 | 57 | 1 |
| Transcription elongation factor SPT6 | G7PTX9 | *Macaca fascicularis* | 198.967 | 1 | 36 | 1 |
| Transketolase | Q5R1W6 | *Pan troglodytes* | 67.797 | 27 | 284 | 9 |
| Tropomyosin alpha-3 chain | P06753 | *Homo sapiens* | 32.93 | 17 | 73 | 2 |
| Tropomyosin beta chain | P07951 | *Homo sapiens* | 32.831 | 8 | 71 | 1 |
| Tubulin beta chain | F6QAX2 | *Macaca mulatta* | 50.4 | 2 | 40 | 1 |
| Tumor necrosis factor receptor superfamily member 6 | P25445 | *Homo sapiens* | 37.708 | 5 | 25 | 1 |
| Uncharacterized protein | F7E526 | *Macaca mulatta* | 59.936 | 12 | 107 | 5 |
| Uncharacterized protein | F6RSN8 | *Macaca mulatta* | 54.523 | 3 | 51 | 1 |
| WD repeat-containing protein 1 | O75083 | *Homo sapiens* | 66.152 | 14 | 78 | 4 |
| Zymogen granule protein 16 homolog B | Q96DA0 | *Homo sapiens* | 22.725 | 4 | 51 | 1 |
