# Supplementary figures and images for: Salivary proteome of a Neotropical primate: potential roles in host defense and oral food perception
Source: PeerJ. 2020 Jul 22;8:e9489. doi: 10.7717/peerj.9489 (PMC7382365; doi:10.7717/peerj.9489)

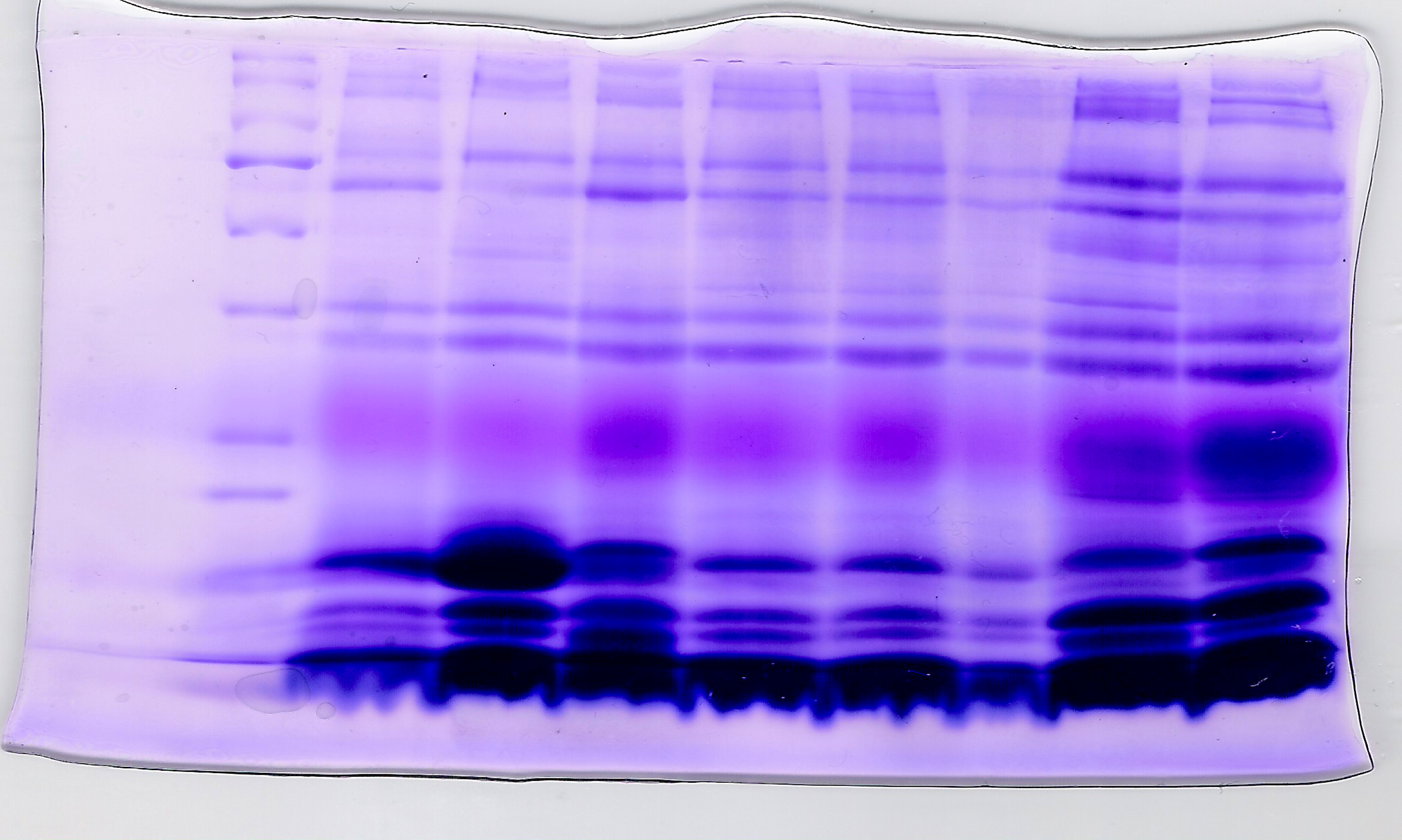

Supplement: Supplemental Information 3 [file peerj-08-9489-s003.jpg]
